# Protocol for a randomised pilot study of a novel Parent–Child Interaction Therapy (PCIT) ‘re-implementation’ intervention

**DOI:** 10.1186/s40814-023-01309-y

**Published:** 2023-05-03

**Authors:** Melanie J. Woodfield, Tania Cargo, Sally Merry, Sarah E. Hetrick

**Affiliations:** 1 Centre for Infant, Child and Adolescent Mental Health, Department of Psychological Medicine, University of Auckland, Auckland, New Zealand; 2 Te Toka Tumai Auckland (Health New Zealand), Auckland, New Zealand; 3 Department of Psychology, University of Auckland, Auckland, New Zealand; 4 Centre for Youth Mental Health, University of Melbourne, Melbourne, USA

**Keywords:** PCIT, Parent–Child Interaction Therapy, Conduct problems, Evidence-based treatment, Feasibility, Time-out, Re-implementation, Implementation

## Abstract

**Background:**

Despite a number of clinicians having been trained in Parent–Child Interaction Therapy (PCIT) in Aotearoa/New Zealand, few are regularly delivering the treatment, with barriers to use including a lack of suitable equipment and lack of professional support. This pragmatic, parallel-arm, randomised, controlled pilot trial includes PCIT-trained clinicians who are not delivering, or only rarely utilising, this effective treatment. The study aims to assess the feasibility, acceptability and cultural responsivity of study methods and intervention components and to collect variance data on the proposed future primary outcome variable, in preparation for a future, larger trial.

**Methods:**

The trial will compare a novel ‘re-implementation’ intervention with a refresher training and problem-solving control. Intervention components have been systematically developed to address barriers and facilitators to clinician use of PCIT using implementation theory, and a draft logic model with hypothesised mechanisms of action, derived from a series of preliminary studies. The intervention includes complimentary access to necessary equipment for PCIT implementation (audio-visual equipment, a ‘pop-up’ time-out space, toys), a mobile senior PCIT co-worker and an optional weekly PCIT consultation group, for a 6-month period. Outcomes will include the feasibility of recruitment and trial procedures; acceptability of the intervention package and data collection methods to clinicians; and clinician adoption of PCIT.

**Discussion:**

Relatively little research attention has been directed at interventions to resurrect stalled implementation efforts. Results from this pragmatic pilot RCT will refine and shape knowledge relating to what it might take to embed the ongoing delivery of PCIT in community settings, providing more children and families with access to this effective treatment.

**Trial registration:**

ANZCTR, ACTRN12622001022752, registered on July 21, 2022.

**Supplementary Information:**

The online version contains supplementary material available at 10.1186/s40814-023-01309-y.

## Contributions to the literature

- Implementation efforts in real-world settings often involve training cohorts of clinicians in an EBT and hoping they will use it in their work—an approach that typically has limited success.
- There are seldom efforts to ‘re-implement’ or resurrect a stalled implementation initiative with trained clinicians who are not (or are only rarely) using the EBT, even where clinicians view the EBT as effective and acceptable.
- This randomised, controlled, pilot study will assess the feasibility, acceptability and cultural responsivity of a new Parent–Child Interaction Therapy (PCIT) re-implementation intervention. It compares refresher training and a package of re-implementation supports (with each component selected deliberately to address identified barriers) with refresher training alone.

## Background

A degree of challenging behaviour such as defiance, non-compliance or aggression is developmentally normal in childhood. However, for a proportion of children, these difficulties are sustained beyond early childhood and may become adversely impactful on child wellbeing and family functioning. Clinical-level childhood conduct problems are among the most common mental disorders formally diagnosed in children under 7 years internationally, represent around half of all childhood psychopathology, are one of the most common reasons for children to be referred to mental health services and were the leading cause of burden (within all mental disorders) for 0–14-year-old children in the most recent Global Burden of Disease study [1–4]. Indigenous children are often over-represented in prevalence data, with complex factors, including the experience of colonisation, racism and subsequent socioeconomic adversity, likely to be relevant. This has been described in relation to Indigenous Māori tamariki (children) and whānau (families) in Aotearoa/New Zealand (see [5–7]).

Evidence-based treatments for childhood conduct problems typically involve ‘parent training’ approaches, based on social learning theory and attachment principles [2]. Parent–Child Interaction Therapy (PCIT; [8]) is one example and is somewhat distinctive from other programmes in its use of in vivo (live) clinician coaching of parents with their 2.5 to 7-year-old children, typically utilising a one-way mirror and a discrete ear-piece for the parent for approximately 12–20 weekly sessions [8]. Meta-analyses have shown that PCIT is effective in reducing conduct problems in children, improving child compliance, reducing parent stress and improving parent emotion regulation and reflective functioning [9–12]. There is mixed evidence relating to PCIT’s effectiveness with indigenous populations [13].

### Implementation of PCIT in Aotearoa/New Zealand

To become accredited in PCIT with PCIT International (www.PCIT.org), a registered masters-level clinician (e.g. psychologist, psychiatrist, social worker, therapist) must initially undertake a 40-h training in the approach, followed by fortnightly PCIT consultation and observation of practice until at least two PCIT cases have been completed successfully. In Aotearoa/New Zealand, approximately 135 clinicians have participated in the 40-h PCIT training since its introduction to the country in 2010 (T.Cargo, personal communication, May 2022) and 13 (i.e. 9.6%) are accredited as PCIT therapists with PCIT International (www.pcit.org; accessed 6 May 2022), this excludes the national trainer and two within-agency trainers.

Existing research suggests that PCIT is viewed as an effective and acceptable intervention to both PCIT-trained clinicians in Aotearoa/New Zealand [14, 15], to Indigenous Māori parents when delivered ‘by Māori, for Māori’ [13], and to families accessing a public mental health service in Aotearoa/New Zealand [16]. However, a 2019 survey of PCIT-trained clinicians in Aotearoa/New Zealand found that—even where the clinician was using PCIT in their work—the average number of families seen per week for PCIT, per clinician was 1.03 [15]. A subsequent 2021 survey of PCIT-trained clinicians found that the median (IQR) typical number of clients seen for PCIT per week was 0 (0 to 1) in Aotearoa/New Zealand, compared to 2 (2 to 3) in Australia, a statistically significant difference [14].

Clearly, despite clinicians having received intensive training in an evidence-based treatment that is acceptable to them, encountering barriers in the real world of service delivery may result in relatively few delivering PCIT to a few families. This research-to-practice gap is a product of complex determinants and is well recognised internationally [17–21].

Implementation science is a relatively new field of research, with a focus on developing knowledge around strategies that are effective for embedding or implementing already-proven treatments into usual care environments [21, 22]. The design of many implementation interventions has historically been driven by the ‘It Seemed Like A Good Idea At The Time’ (ISLAGIATT) principle, and there have been calls for more systematic and theory-driven development of implementation interventions, in order to improve their effectiveness and specificity [23]. The Theoretical Domains Framework of behaviour change is a compilation of domains which condense and describe modifiable factors that may influence clinician behaviour [24]. The 14 domains have been mapped onto three central factors: an individual’s Capability (physical and/or psychological), Opportunity (social and/or physical) and Motivation (automatic and/or reflective) to perform a particular behaviour [24, 25]. This COM-B model can be used to understand influences on clinician behaviour and facilitate the systematic design of implementation interventions [23]. Incorporating models such as the COM-B may also more readily enable the outcomes of these interventions to be linked to mechanisms of action—another important direction for the field [26].

### Proposed intervention

Our recent systematic review of existing interventions to support the implementation of PCIT suggested that PCIT implementation research has been disproportionately targeted at training initiatives [27]. Rather than prioritising the training of more clinicians in PCIT (though this remains important), our intention is to better understand what it would require for already-trained clinicians to begin (or resume) implementing PCIT in their practice. We have described this as ‘re-implementation’, which we defined as ‘resuming implementation, implementing again and/or implementing differently’ [14]. Relatively little research attention has been given to reinvigorating unsuccessful programme implementation, possibly due to pragmatic considerations such as grant funding expiring after initial implementation efforts (i.e. initial training of clinicians). Although there have been recent indications of interest in the area—which has been described as ‘implementation, interrupted’ in Uganda [28].

In our preliminary work, we sought to understand the barriers and facilitators of clinician use of PCIT in Aotearoa/New Zealand, following the systematic method advocated by Atkins [23]. We acknowledge that there will be a proportion of clinicians who do not use PCIT post-training for compelling reasons, such as cessation of clinical practice, or those to whom the approach is not acceptable. Our interest is in those clinicians to whom PCIT is acceptable, who may have encountered barriers which have impeded their initial desire to incorporate PCIT in their practice. Through engaging with PCIT-trained clinicians in surveys and focus groups, we have identified that these barriers include the lack of access to suitable equipment to support the delivery of PCIT (for example, audio-visual equipment) and families being unable to easily attend clinic-based sessions (traditionally held during working hours in a fixed location) [14]. Facilitators of PCIT’s use included access to a suitable room or clinic space, the ability to co-work PCIT cases with another clinician and having other PCIT-trained colleagues in the workplace [14].

The package of re-implementation supports described in this paper has been specifically designed to address these identified barriers and is based on a preliminary logic model of mechanisms of action from our earlier work (paper in preparation). In summary, we hypothesise that the difficulties accessing equipment (physical opportunity) and lack of a co-worker and concerns from colleagues and administrators about components of PCIT (social opportunity) work to decrease motivation to implement PCIT (the behaviour). Introducing a PCIT co-worker and regular group consultation sessions is hypothesised to improve psychological capability and social opportunity, and providing equipment (time-out tents and audio-visual equipment) is hypothesised to increase both physical and social opportunity; both of which influence motivation to implement PCIT (the behaviour). The package of supports is designed to be flexibly utilised, for example outside of traditional clinic hours or in a family’s home, and we anticipate that this will better facilitate client attendance.

Piloting this novel package of supports will allow us to refine the development of the re-implementation intervention [29], and our preliminary logic model, in order to increase the specificity of mechanisms of action to test in a future trial [30].

### Study aims

The primary objective of this pilot study is to assess the feasibility of conducting a RCT. In the future, this fully powered RCT will assess the effects of the provision of a re-implementation intervention on the adoption of PCIT by clinicians who are not—or are rarely—using PCIT in their practice. The current feasibility trial will identify any factors that may detract from our ability to achieve this aim in the full trial.

This trial aims the following:

1. Pilot the recruitment and trial procedures to assess recruitment rates, assess the characteristics of clinicians who enrol in the trial, assess whether the idea of randomisation is acceptable to participants.
2. Identify any specific cultural factors that may influence recruitment, data collection methods or sampling frameworks and determine the acceptability of the reimplementation intervention for Māori participants.
3. Assess the acceptability of the re-implementation package to clinician participants, in order to refine the components of the PCIT re-implementation intervention.
4. Pilot the use of a series of online self-report surveys as a data collection method for assessing the effects of the re-implementation intervention on clinician capability, opportunity and motivation.
5. Explore potential effects of the PCIT re-implementation intervention on clinician adoption of PCIT. This will include identifying the variability of the proposed primary outcome variable at the end of the intervention, to calculate an appropriate sample size for a definitive RCT.

## Methods

### Trial design

The study will involve a pragmatic, randomised, controlled parallel-arm pilot trial of a re-implementation intervention. We have elected to randomise at the level of the clinician, as our primary focus is understanding the potential effectiveness of the re-implementation package. Also, while context exerts a significant influence on implementation [31], individual behaviour change remains at the core of implementation success, even where there is significant influence from organisational factors [32]. In fact, individual factors such as attitudes may be more predictive of implementation than organisational factors, and individual-level barriers may be more amenable to intervention [32]. Also, we are interested in understanding where (i.e. which clinical settings) clinicians choose to adopt PCIT as a result of the intervention, in order to inform the design of the future trial.

### Participants

#### Inclusion criteria

Clinicians must have completed a recognised 40-h initial training in PCIT no earlier than 2010 (when PCIT was introduced to Aotearoa/New Zealand) and be registered to practise in Aotearoa/New Zealand at the time of participation in the trial. Having been eligible for the PCIT initial training implies that included clinicians will be allied health and medical clinicians with a Master’s degree or equivalent (i.e. psychologists, psychiatrists, social workers, psychotherapists, occupational therapists and nurses). Eligible clinicians are not required to be employed in a clinical role and may be in an administrative or managerial role, as it is possible that they may elect to adopt PCIT in a part-time private practice context. Where the clinician is already seeing a full caseload of PCIT clients (our preliminary research suggests this is unlikely), they will remain eligible for inclusion as we are interested in whether the provision of additional supports might influence the nature and quality of implementation. For example, the PCIT treatment protocol recommends the use of a time-out room in the Parent-Directed Interaction phase, and clinicians have indicated that this requirement can be problematic, which at times results in this phase being omitted or adapted [14].

#### Exclusion criteria

Clinicians who are based outside of the Tāmaki Makaurau/Auckland region will be excluded from this pilot trial, due to resource constraints. The mobile co-worker/consultant will be Tāmaki Makaurau/Auckland-based in the pilot trial.

#### Setting and proposed recruitment

Approximately 135 clinicians have received the 40-h initial PCIT training in Aotearoa/New Zealand since it was introduced in 2010. Participants will be recruited from a group of approximately 70 clinicians who are located in Tāmaki Makaurau/Auckland—Aotearoa/New Zealand’s largest city. The authors have an existing database of eligible clinicians, compiled for an earlier research project [14]. We will use the cultural practices of whanaungatanga to obtain details for those Māori clinicians who were unable to be contacted in the earlier study. Eligible PCIT-trained clinicians will then be approached via email, with a flyer inviting their attendance at a 1-h virtual information session. A second reminder email will be sent 2 weeks after the first approach. If an email address is unavailable, the first author will search publicly available information online and eligible clinicians will be approached by phone if necessary. If the clinician identifies as Māori, this phone call will be made by a Māori research team member to support culturally responsive practice. The flyer and Participant Information Sheet will also be distributed by Whāraurau—a government-funded child and adolescent workforce development agency—to those clinicians who received training in PCIT through funding from the Ministry of Health.

At the information session, clinicians will be provided with information about what the trial will involve, the risks and benefits of participation, and will have the opportunity to ask questions. They will be asked to submit an expression of interest in receiving further information, confirming their details via a very brief online Qualtrics survey at the end of the session. If unavailable for the information session, clinicians can also submit an expression of interest independently. Those clinicians who choose not to express interest in proceeding to the trial will be asked (via the Qualtrics survey) for a brief reason why they do not want to, or are not able to, proceed. This information will be used to inform recruitment processes for the future trial including specific tikanga Māori practices.

Clinicians who express interest will be emailed a full Participant Information Sheet (PIS) and consent form. Those who consent to participate will be invited to a two-day PCIT refresher training in Tāmaki Makaurau/Auckland (refer below).

#### Randomisation and blinding strategies

All participants will have been recruited and will have participated in the two refresher training days (refer ‘Study Treatments’, below) prior to random allocation to intervention and control groups. Allocation to groups will occur at the end of the second day of refresher training. A random allocation sequence will have been generated by the study statistician (using a simple 1:1 randomisation with no restrictions), who will contact participants via email with their group allocation. Participants will be asked not to share the email or reveal or discuss the condition to which they have been allocated. We will undertake a test of blinding at the end of the intervention (i.e. will ask participants which condition they believed themselves to have been allocated) in order to determine whether blinding is feasible in a future trial.

The first author will be delivering some of the re-implementation supports and will therefore be unable to be blinded. The remainder of the research team, particularly outcome assessors, will be blinded.

#### Other considerations

Participants in both the intervention and control groups are free to undertake additional professional development or supervision at their discretion. At the conclusion of the trial, these additional activities will be recorded.

Participants will be free to withdraw from both intervention and follow-up activities. They will be sent an email from a member of the research team (not the first author, who will be delivering aspects of the intervention) and asked to provide a brief reason for their withdrawal, as this information is useful to inform recruitment and retention of participants in the future trial. The point of withdrawal will be documented, and participants will be advised in the consent form that data gathered up until the point of withdrawal will remain available to the investigators for analysis.

### Intervention and control conditions

#### All participants

All participants will undertake a 2-day refresher training, the content of which is informed by PCIT International’s ‘recalibration’ training, by previous research into implementation barriers encountered by clinicians in Aotearoa/New Zealand and culturally responsive practices. This complimentary, catered training will be delivered on two successive Saturdays, to facilitate clinician attendance regardless of their current employment context. It will be co-delivered by an experienced Māori PCIT provider and a senior PCIT trainer in Tāmaki Makaurau/Auckland and will only be delivered virtually if absolutely necessary due to COVID-19 restrictions.

Incorporated within the two training days, *all* participants will receive the following enhanced supports:

- A collection of resources to support delivery, including detailed virtual delivery resources and demonstration of these. Access to a Dropbox with all resources and materials.
- A voucher for a complimentary pack of 25 Eyberg Child Behaviour Inventory (ECBI; [33]) questionnaires to use with future PCIT clients, and a complimentary PCIT treatment protocol in the event that this has been misplaced since their initial PCIT training.
- Facilitated discussion and problem-solving sessions around using PCIT in various contexts including private practice should participants choose to do this. For example, what to charge for PCIT, how to obtain referrals and where suitable clinic rooms are located.
- Discussion, facilitated by a Māori PCIT provider, relating to how to deliver PCIT in a culturally responsive way.

After the two training days, *all* participants across both groups will be offered optional complimentary weekly 1-h group PCIT-oriented consultation with a senior PCIT clinician, for the 6-month trial period.

#### Intervention

Specific components of the intervention are provided in Table 1, according to the TIDieR checklist [34]. In summary, clinicians allocated to the intervention condition will receive the following:

- A pack of suitable PCIT *toys* to use with children and families, which will be provided free of charge.
- Access to a mobile *co-worker*/‘PCIT partner’ who can be booked through an online booking system free of charge at any time. This senior PCIT clinician will be available to join a client session to support the clinician (the clinician is responsible for obtaining client consent for this to occur), or to plan or debrief sessions, as requested by the clinician, and in any location of the clinician’s choice. We will recruit a senior Māori PCIT clinician to this role, as Māori are the Indigenous people of Aotearoa/New Zealand and a number of PCIT-trained clinicians are Māori (14.8% in our 2021 survey).
- Access to portable, relocatable *audio-visual equipment,* including a high quality digital remote camera and monitor, which allows viewing of the clinic room from a nearby room and a Bluetooth earpiece to facilitate clinician coaching of the parent(s) from the nearby room.
- Access to an in-room *time-out pop-up cubicle* to be used if the child requires a brief time-out, that is transportable to the participant clinician’s clinic room of choice.

**Table 1:** Re-implementation intervention components, described using the TIDieR checklist

Brief name	Re-implementation support package for PCIT-trained clinicians
Why	PCIT is an effective treatment for childhood conduct problems, and a number of clinicians have received a five-day initial training in the approach since it was introduced to Aotearoa in 2010. Our earlier work suggests that, despite PCIT-trained clinicians viewing PCIT as effective and acceptable, relatively few are using it in their work, and those who are using it, are seeing a small number of families. The majority of PCIT-trained clinicians describe having encountered barriers to its use, which include lacking suitable equipment, and families having difficulty accessing clinic-based sessions. Facilitators of PCIT’s use included access to a suitable clinic room, and the ability to co-work PCIT cases with another clinician. These earlier studies have shaped the selection of intervention components, which have also been informed by the COM-B behaviour change theory
What	**Materials** • Participating clinicians will have access to a Dropbox folder of PCIT-related resources, including research articles, slides from relevant presentations, handouts and worksheets. These have been produced by a number of different PCIT researchers and clinicians • Clinicians will be provided with a toy package of approximately $NZD700 value, containing toys selected by two senior PCIT clinicians (Māori and non- Māori) as being suitable for use in PCIT • Two robust Voyager Compact travel bed tents from Safespaces UK (www.safespaces.co.uk) will be available for clinicians to book, to use as portable time-out spaces for children. Setup support will be provided to clinicians • Audio-visual equipment will include a ‘baby monitor’ kit, to allow remote camera viewing and audio from the adjacent room, by a clinician located in a separate space to the parent and child. A basic mobile phone and earpiece worn by the parent will allow clinicians to discretely provide coaching out of the child’s hearing **Procedures** • Refresher training content will be compiled and delivered by two senior PCIT-trained clinicians (Māori and non-Māori), one of whom is a Within-Agency Trainer for PCIT. It will include pre-existing video content from the UC Davis PCIT web course, and the Auburn University PCIT continuing education videos. The two trainers will also facilitate discussions and problem-solving sessions relating to how to deliver PCIT in a culturally responsive way, navigating client or colleague concerns relating to the use of time-out, and other topics that have been identified in our earlier research as impeding or influencing implementation. These discussions will be informed by the trainers’ own research and clinical experience
Who provided	The refresher training will be delivered by two PCIT-trained clinical psychologists, one of whom is Māori, and one of whom is a PCIT International accredited Within-Agency Trainer. The co-worker is an accredited PCIT provider, a Māori clinical psychologist. The weekly PCIT consultation groups will be facilitated by the first author, with support from the co-worker, who will also attend
How	Refresher training will be delivered in person. Weekly PCIT consultation sessions will be delivered in a hybrid format, where clinicians can join via Zoom or in person, according to their preference. Co-working contacts may involve the co-worker joining a client session (client consent having been obtained by the participating clinician in advance) or in person contact
Where	Refresher training will occur at a University clinic training room, over two successive Saturdays. Co-working will occur at a location of the clinician’s choice (e.g. their office, a Zoom call)
When and How Much	Refresher training ‘dose’ is fixed at two 8-h days, that will be scheduled approximately 6 weeks after the trial information sessions. PCIT consultation groups will occur weekly, for 1 h, across the 6-month trial period. Co-worker contacts are not fixed and are unlimited, and the uptake of this component is of interest as a study outcome
Tailoring	The re-implementation intervention will not be adapted or personalised by the research team; however, participating clinicians will select additional components that are of interest or relevance to them, using the online booking system. These additional components include (1) audio-visual equipment, (2) time-out pop-up tents, and (3) access to a co-worker
Modifications	N/A for protocol
How well	Intervention fidelity and adherence will not be assessed

These components will be available immediately after the refresher training, for the 6-month trial period. The toy package will be held by the individual participating clinicians, and all other components booked through an online booking system.

Given that there is uncertainty as to the extent to which an in vivo consultant aids clinician implementation of PCIT relative to the provision of equipment, we considered separating these components and investigating the influence of each on implementation. However, in practice, the co-worker (a senior PCIT clinician) is likely to discuss equipment, session logistics and clinic room setup in their interactions with the clinician participants while co-working sessions. As such, and given the pragmatic nature of this pilot trial, we have elected to combine these.

We also considered fitting-out a specialised clinic room in Tāmaki Makaurau/Auckland; however, one location may not be convenient for all participants, who are located throughout the city. Also, one centralised clinic room is less flexible and less scalable than a collection of modifiable supports. For example, a participating clinician may choose to implement PCIT in the client’s home, or in their existing workplace/agency, and/or may only require one or two elements from the package.

#### Comparison

In designing the control condition, our intention was to provide a more active condition than simply (re)training as usual. Given that clinicians have already trained in PCIT previously, we were mindful of enhancing the content so as to make participation worthwhile for clinicians and to justify the costs associated with replicating the training. As such, and as outlined in ‘All participants’ above, participants in the control condition will receive enhanced refresher training, which includes problem-solving and facilitated planning and implementation discussions, along with basic resources to aid implementation, such as ECBI questionnaires. They will also receive optional complimentary weekly PCIT-oriented consultation.

**Fig. 1:**
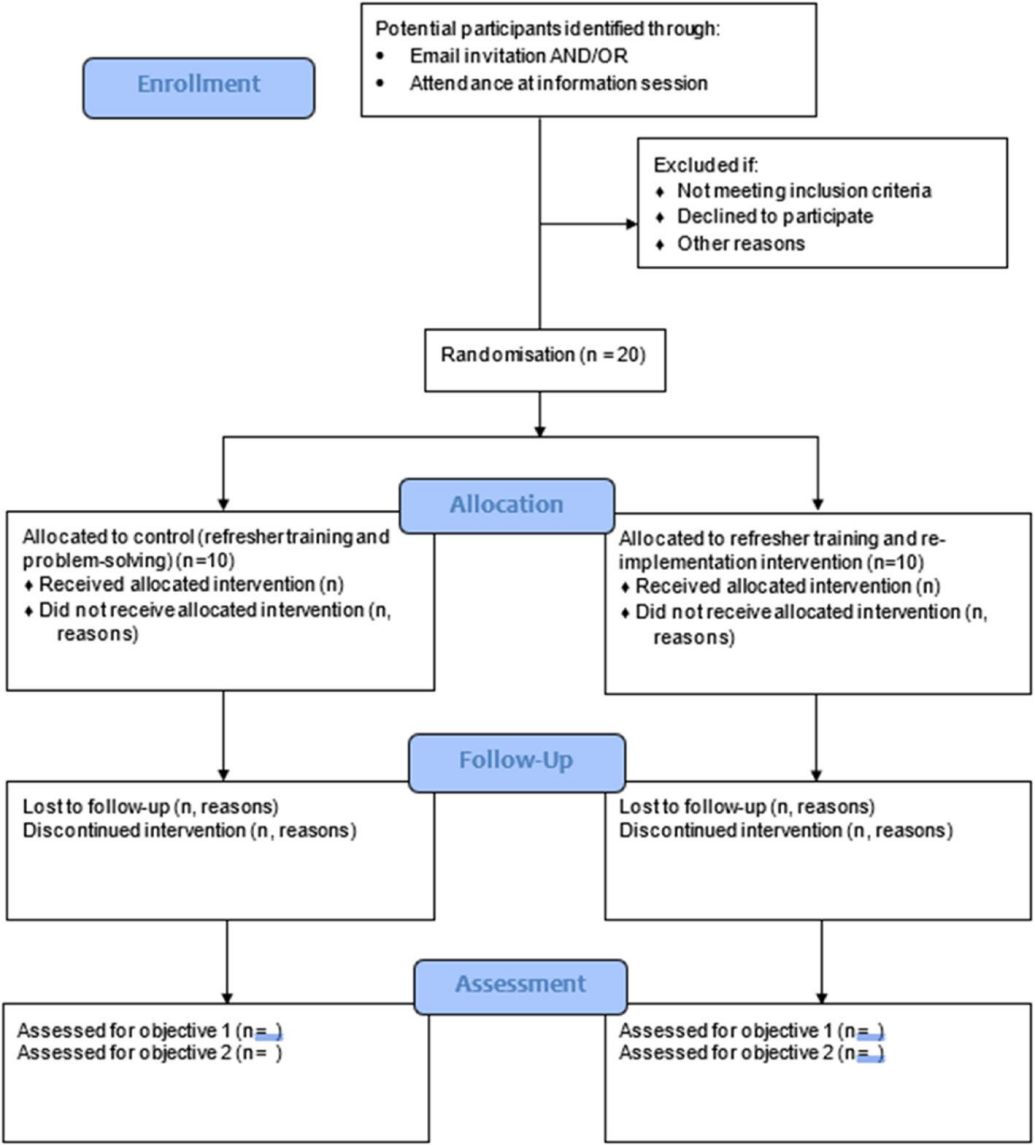
CONSORT flow diagram

Figure 1 represents the CONSORT flow diagram, and the CONSORT checklist is provided as an Additional file [35].

### Outcomes and measures

Baseline data will be collected from all participants, via a Qualtrics survey prior to allocation being revealed. The re-implementation intervention will be available for a period of 6 months to the intervention group. Some aspects of the re-implementation intervention occur at one point in time (e.g. the refresher training) and others continue across the trial period (e.g. access to equipment and co-worker, consultation). All aspects of the intervention will be contained within a 6-month period and will cease at that point. Table 2 provides a summary of outcomes and measures.

**Table 2:** Summary of outcomes, measures and time points

		**0**	**1**		**2**		**3**	
**Method**		**Pre-trial information session**	**Baseline (beginning of day 1 of refresher training)**		**Monthly throughout trial (begins 1-month post-randomisation)**		**End of trial (6 months post randomisation)**	
			**Control**	**Intervention**	**Control**	**Intervention**	**Control**	**Intervention**
**Acceptability of trial processes and data collection methods**								
Study-specific Qualtrics questionnaire	Acceptability of trial processes (incl. to those choosing not to enrol)	X					X	X
	Number of participants enrolled in trial	X						
	Number of participants completing trial						X	X
	Response rates to study questionnaires				X	X		
	Test of blinding						X	X
**General data**								
Study-specific Qualtrics questionnaire	Age and gender		X	X				
	Ethnicity		X	X				
	Professional discipline		X	X				
	Service/setting characteristics		X	X				
	Role characteristics (e.g. other demands, FTE worked)		X	X				
	Professional development activities (e.g. training and supervision) undertaken in six month trial period – in addition to trial intervention components						X	X
**Adoption**								
Study-specific Qualtrics questionnaire	Number of families provided with PCIT in past 1 week		X	X	X	X	X	X
**Implementation determinants**								
Theoretical Domains Framework Questionnaire [36] (via Qualtrics)	61 items, 7-point Likert scales		X	X			X	X
Capability, Opportunity, Motivation to use PCIT (based on [37]) (via Qualtrics)	3 items, 10-point Likert scales				X	X		
**Acceptability of intervention components**								
Online booking system data	Usage of audio-visual equipment, time-out tents, co-worker					X (weekly)		
	Attendance at group consultation sessions				X (weekly)	X (weekly)		
Semi-structured interviews							X	X

#### Primary outcome

The primary objective of this pilot study is to assess the feasibility of conducting a RCT. In future, this fully powered RCT will assess the effects of the provision of a re-implementation intervention on the adoption of PCIT by clinicians who are not—or are rarely—using PCIT in their practice. The current feasibility trial will identify any factors that may detract from our ability to achieve this aim in the full trial. Primary outcomes are (1) the number of clinicians who enrol in the trial and (2) the number of clinicians who complete the trial.

- (1) Recruitment processes will be evaluated by assessing the number of clinicians who provide consent to participate in the trial—this will be determined by an audit of study enrolment logs at baseline (beginning of day 1 of PCIT refresher training).
- (2) Trial procedures will be evaluated by assessing the number of clinicians who complete the trial, as determined by an audit of study logs at the end of the trial (6 months post-randomisation). When a clinician withdraws from the study, the point of withdrawal and reason for withdrawal will be recorded.

#### Secondary outcomes

Acceptability of trial processes to participants will be measured by a study-specific survey following the pre-trial information session and at the end of the trial. Those who choose *not* to enrol in the trial following the information session will also be asked to complete the study-specific survey as to their reasons for non-participation, and a frequency count of responses to these multiple-choice items will be undertaken.

Clinician self-reported Capability, Opportunity and Motivation to use PCIT (drawn from COM-B theory; [23]) will be measured by Likert scale items within a monthly self-report survey, developed for the purposes of this trial and delivered via Qualtrics.

Acceptability of the monthly surveys as a data collection method, and the timing and frequency of these, will be measured by response rates. Also, a post-trial semi-structured phone interview will incorporate Likert scales and seek participants’ ratings of the acceptability of the monthly surveys as a data collection method.

Acceptability of each aspect of the re-implementation intervention will be assessed by participant uptake of the components. As an online booking system will be used to coordinate the loan of (1) the audio-visual equipment, (2) the time-out cubicle and (3) the mobile co-worker, a frequency count of weekly bookings will be used as a proxy measure for the acceptability of these items.

Also, in a post-trial semi-structured phone interview, participating clinicians will be asked to rate the acceptability of the components of the re-implementation package on a series of Likert scales. Acceptability of group consultation sessions will be assessed by participant attendance rates, measured by an audit of session attendance registers.

Clinician adoption of PCIT will be assessed by clinician self-report of the number of unique families to whom PCIT was delivered in the most recent representative 1-week period at 6 months post-randomisation and compared to baseline (via the study-specific monthly survey). Given that PCIT sessions are typically held weekly, this is expected to provide a valid measure of clinician PCIT adoption.

Acceptability of the recruitment and trial processes to Māori clinicians will be assessed by multiple-choice items within a study-specific questionnaire following the pre-trial information session and at the end of the trial. Māori clinicians choosing not to enrol in the trial will also be offered the opportunity to speak kanohi-ki-te-kanohi (face to face) or via phone with a Māori research team member, to discuss their impressions and any concerns about trial recruitment processes and/or methodology that contributed to their decision not to participate, and this qualitative data will be recorded.

### Statistical considerations

#### Sample size and justification

As this is a feasibility study, sample size calculations are not required, and a formal power calculation is not appropriate, given that the primary outcome for this pilot is feasibility [38]. Instead, sample sizes for feasibility studies should be based on pragmatic considerations [39]. However, for the RCT, it is envisaged that the primary outcome will be *adoption*: clinician self-report of the number of unique families to whom PCIT was delivered in the most recent representative 1-week period at 6 months post-randomisation, compared to baseline. These data will be collected.

Relevant pragmatic considerations included the size of the group of PCIT-trained clinicians thought to be practicing in the Tāmaki Makaurau/Auckland region, excluding the research team (approximately 70). Also, the budget for the research project will only support limited co-worker hours, and the purchase of limited time-out pop-up cubicles and limited audio-visual equipment and toy packages. Given these practical constraints, we hope to recruit 10 participants per condition or 20 overall. We are interested in evaluating whether the proposed recruitment methods will be effective in a future, larger trial. While we will aim to recruit this number, our ability to do so will provide valuable data around our ability to recruit sufficient participants for a fully powered trial using the described methods.

#### Progression criteria

The following criteria will be used to assess whether it is feasible to progress to a fully powered randomised controlled trial in the future.

1. Clinician recruitment rate: study is considered feasible if at least 20 clinicians enrol in the trial. Given that we have contact information for 70 PCIT-trained clinicians in the region (i.e. the population of interest), we consider this number to be reasonable.
2. Clinician trial completion rate: study is considered feasible if there is a maximum of 20% attrition.
3. Acceptability of surveys as a data collection method: study is considered feasible if the average survey response rate (across the baseline, monthly and end-of-trial surveys) is at least 80%.

#### Statistical methods

The purpose of pilot studies is not to test hypotheses, and inferential statistics are not required [39]. Descriptive statistics will predominantly be used to summarise participant characteristics and outcome measures. While it is acknowledged that the smaller sample size associated with pilot studies can provide unstable effect size estimates, we intend to carry out some exploratory analyses alongside descriptive statistics for clinician adoption of PCIT, by way of a two-sample *t* test. We will note—but not statistically account for—observed differences between groups at baseline. Measures of variance on this outcome variable will be used to inform the design of a definitive RCT.

## Discussion

This pilot trial will help determine whether it is feasible to carry out a randomised controlled trial of the proposed re-implementation intervention and will assist with refining the intervention itself. The proposed study represents a systematically developed, theory-driven intervention, with components selected deliberately with hypothesised mechanisms of action in mind and thus is an advance on previous implementation studies [23, 26]. Whilst modest in scale, pilot or feasibility studies such as this are important ‘bricks in the wall’ of understanding complex implementation challenges and shaping and refining novel interventions such as the ‘re-implementation’ intervention described here [29].

The study is intentionally pragmatic and provides easily accessible tangible supports for implementation, which had previously been difficult to access for clinicians [14]. This pilot trial will allow us to assess the feasibility of delivering a novel re-implementation intervention to support the delivery of an evidence-based treatment, with the ultimate aim of making PCIT more available to children and families in need.

## Supplementary Information

**Additional file 1.** CONSORT (2010) checklist.

## Data Availability

Not applicable.
